# Exosomes derived from mesenchymal stem cells repair a Parkinson’s disease model by inducing autophagy

**DOI:** 10.1038/s41419-020-2473-5

**Published:** 2020-04-27

**Authors:** Hong-Xu Chen, Fu-Chao Liang, Ping Gu, Bian-Ling Xu, Hong-Jun Xu, Wen-Ting Wang, Jia-Yang Hou, Dong-Xiao Xie, Xi-Qing Chai, Sheng-Jun An

**Affiliations:** 1 Department of Neurology, the First Hospital of Hebei Medical University, No. 89 Donggang Road, Yuhua District, Shi Jiazhuang, 050031 Hebei China; 2 Hebei Provincial Engineering Laboratory of Plant Bioreactor Preparation Technology, No. 326 Xinshi south Road, Qiaoxi District, Shi Jiazhuang, 050090 Hebei China; 3 Research Center, Hebei University of Chinese Medicine, No. 326 Xinshi south Road, Qiaoxi District, Shi Jiazhuang, 050090 Hebei China; 4 Department of Gynecology, the Second Hospital of Hebei Medical University, No. 215, HePing West Road, Shi Jiazhuang, 050000 Hebei China; 5 Department of orthopaedic, Third hospital of Hebei Medical University, Shi Jiazhuang, 050000 Hebei China

**Keywords:** Blood-brain barrier, Parkinson's disease, Mesenchymal stem cells, Parkinson's disease, Stem-cell research

## Abstract

Parkinson’s disease (PD) is a progressively debilitating neurodegenerative condition that leads to motor and cognitive dysfunction. At present, clinical treatment can only improve symptoms, but cannot effectively protect dopaminergic neurons. Several reports have demonstrated that human umbilical cord mesenchymal stem cells (hucMSCs) afford neuroprotection, while their application is limited because of their uncontrollable differentiation and other reasons. Stem cells communicate with cells through secreted exosomes (Exos), the present study aimed to explore whether Exos secreted by hucMSCs could function instead of hucMSCs. hucMSCs were successfully isolated and characterized, and shown to contribute to 6-hydroxydopamine (6-OHDA)-stimulated SH-SY5Y cell proliferation; hucMSC-derived Exos were also involved in this process. The Exos were purified and identified, and then labeled with PKH 26, it was found that the Exos could be efficiently taken up by SH-SY5Y cells after 12 h of incubation. Pretreatment with Exos promoted 6-OHDA-stimulated SH-SY5Y cells to proliferate and inhibited apoptosis by inducing autophagy. Furthermore, Exos reached the substantia nigra through the blood–brain barrier (BBB) in vivo, relieved apomorphine-induced asymmetric rotation, reduced substantia nigra dopaminergic neuron loss and apoptosis, and upregulated the level of dopamine in the striatum. These results demonstrate that hucMSCs-Exos have a treatment capability for PD and can traverse the BBB, indicating their potential for the effective treatment of PD.

## Introduction

Parkinson’s disease (PD) is a common progressive neurodegeneration disorder and the second most common neurodegenerative disease after AD, with a prevalence of approximately 1% in people over 60 years of age in industrialized countries^1^. There is a crucial unmet need for a neuroprotective drug for patients with PD, particularly considering that the number of affected patients is set to double over the next 20 years, with over 14 million PD cases expected worldwide by 2040^2^. Therefore, diverse novel therapeutic avenues, in particular stem cell approaches, are of high interest.

Neural stem cells, induced pluripotent stem cells, embryonic stem cells, and mesenchymal stem cells (MSCs) have previously been studied for the treatment of PD^3–6^. Human umbilical cord mesenchymal stem cells (hucMSCs) are ideal for use in therapeutic applications because of their multiline age differentiation capability, autologous transplantation feasibility, easy acquisition, and lack of ethical problems^6^. However, only a small proportion of transplanted hucMSCs has been evidenced in target tissue, transplanted hucMSCs have a low survival rate in the host, and vein-transplanted cells may cause capillary embolization and uncontrollable cell division^7,8^. Therefore, the identification of an alternative to replace hucMSCs transplantation with a similar level of repair activity is urgently required.

hucMSCs secrete a large number of growth factors, cytokines, and chemokines, which have multiple implications in the regulation of key biologic processes such as neuroprotection, neurodifferentiation, the reduction of apoptosis, and the regulation of inflammatory processes^9^. These factors include exosomes (Exos), which are cell-derived bilayer lipid vesicles with a diameter of 30–150 nm that were shown to be involved in information exchange and substance transfer between cells^10,11^. The blood–brain barrier (BBB) is relevant to nervous system diseases by often making it difficult for drugs to reach the lesion site and have a therapeutic effect. However, the nano-size of Exos means they readily cross the BBB. Recently, Exos were also found to be a desirable MSC substitution because they fuse with plasma membranes of recipient cells and transport proteins and RNA into these cells, thereby altering their fate^12^. Additionally, several studies demonstrated that hucMSCs-secreted Exos have a protective effect on cardiovascular and cerebrovascular diseases^9,13,14^. However, the role and mechanism of it in PD remains unknown.

Autophagy is a cellular mechanism that recycles materials such as abnormal proteins and damaged organelles to maintain cell survival and homeostasis. The alteration of autophagy may be a key factor contributing to the pathogenesis of PD^15^, and a number of reports showed that increased autophagy has potential therapeutic effects in PD^16,17^. In the present study, we investigated whether and how Exos repair the damage of PD models in vitro and in vivo by regulating autophagy.

## Results

### Typical features of hucMSCs

**Fig. 1:**
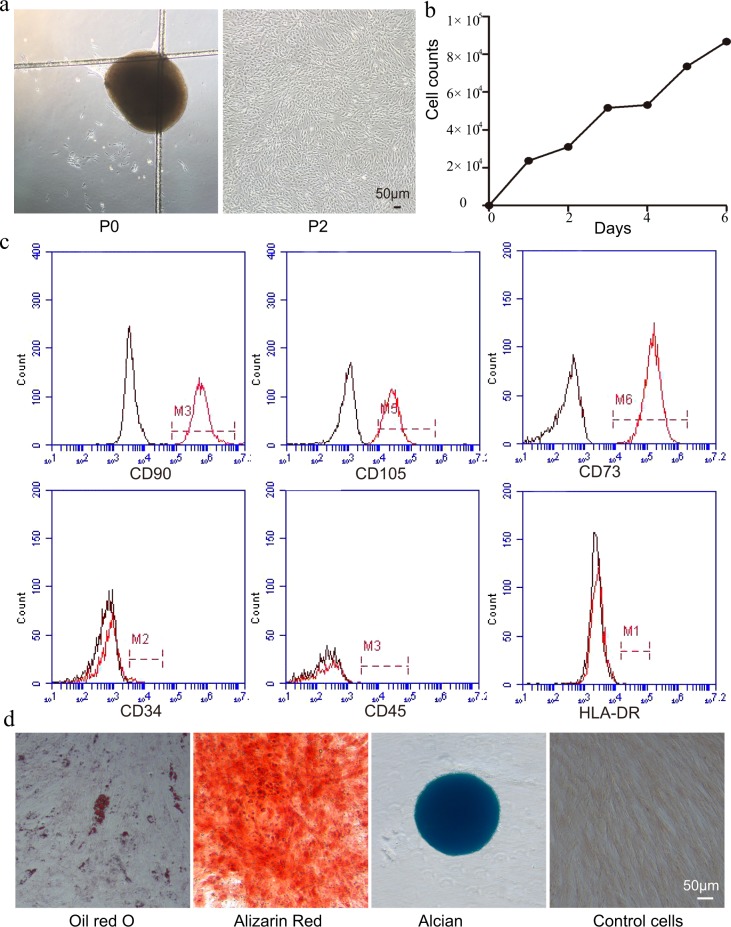
Typical features of hucMSCs. **a** Morphological appearance of hucMSCs. The appearance and growth of hucMSCs colonies after 6 days of culturing (left); after replating the cells took on the appearance of long spindle-shaped fibroblastic cells, began to form colonies and became confluent (right). **b** Growth curve of hucMSCs. The cells grew logarithmically from day 2, and on the third day the growth rate slowed because of the replacement of culture medium. **c** The surface antigens of hucMSCs. The cells were positive for CD90, CD105, CD73 expression and negative for CD34, CD45, and HLA-DR. **d** Results of Oil red O, Alizarin Red, and Alcian Blue detection in cell cultures growing after 3 weeks. Control cells grew in regular medium.

After the initial 6 days of primary culture, spindle-shaped cells adhered to the petri dish. At 10–14 days after plating, they resembled long, spindle-shaped fibroblasts, began to form colonies, and became confluent. After replating, the fibroblast-like cells appeared polygonal or spindly with a long process (Fig. 1a).

Cell growth curves are shown in Fig. 1b. On the second day of growth, cells entered the logarithmic growth phase. On the third day, the growth rate slowed down because of the replacement of culture medium, but it increased rapidly again next day. The cells continued to grow for 6 days, with an S-shaped growth curve, and showed strong self-renewal and reproductive abilities.

Flow cytometry (FCM) demonstrated that cells expressed high levels of CD90, CD105, and CD73, but were negative for CD34, CD45, and human leukocyte antigen (HLA)-DR expression (Fig. 1c). This suggested that the cells were a type of MSC, not haematopoietic cells. After adipogenic, osteogenic, and chondrogenic medium induction, some of the cells had numerous Oil Red O-positive lipid droplets, mineralized nodules (bone) after Alizarin Red staining, and Alcian Blue-positive glycosaminoglycan depositions (cartilage). Non-induced cultures showed no spontaneous adipocyte, osteoblast, or cartilage formation. These results suggest that the cells have the ability to differentiate into osteocytes, adipocytes, and chondrocytes (Fig. 1d).

These findings indicated that the cells isolated in this study were hucMSCs that could be used in subsequent experiments.

### 6-Hydroxydopamine (6-OHDA)-stimulated SH-SY5Y cell viability

6-OHDA is specifically taken up from presynaptic terminals of dopaminergic neurons through the dopamine transporter. In dopaminergic neurons, it is oxidized to produce free radicals, thus leading to neuronal death through mitochondrial dysfunction and oxidative stress^18^. It is a commonly used model drug for PD. Therefore, 6-OHDA-stimulated SH-SY5Y cells and rats were used as PD models in the present study.

**Fig. 2:**
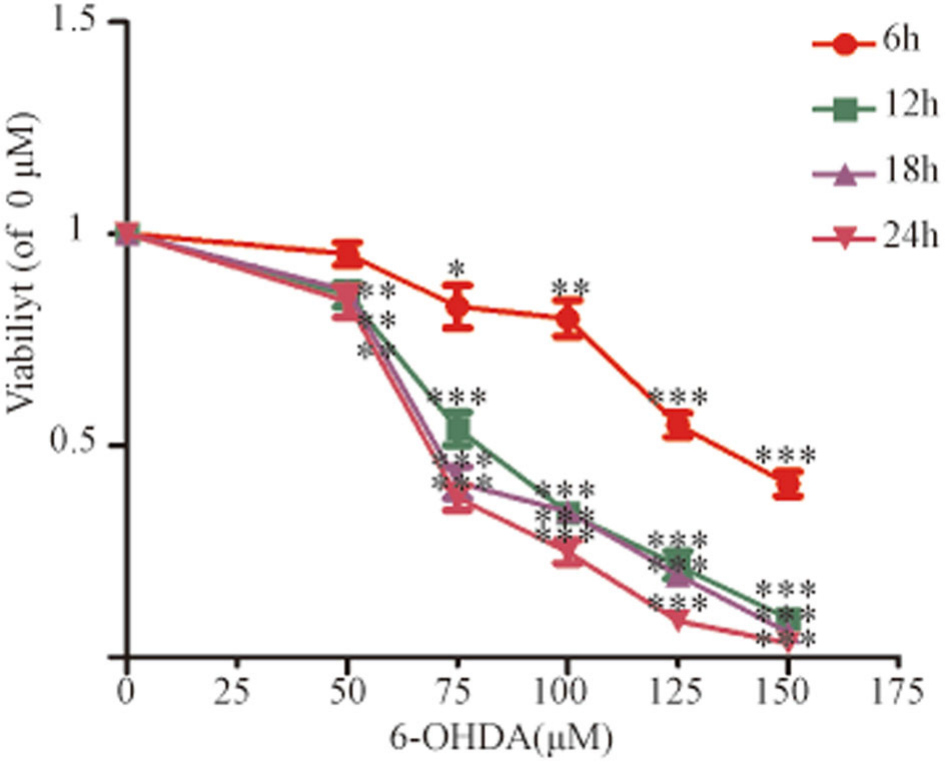
6-OHDA-stimulated SH-SY5Y cell viability. CCK-8 was used to measure SH-SY5Y cell viability after exposure to 50, 75, 100, 125, and 150 µM 6-OHDA for 6 h, 12 h, 18 h, and 24 h. All data represent mean values ± SD. *P < 0.05; **P < 0.01; ***P < 0.001 vs 0 µM 6-OHDA (*n* = 4).

Compared with cells in the 0 µM group, 6-OHDA induced a dose-dependent decrease in cell viability, as measured by the cell counting kit (CCK)-8 assay in SH-SY5Y cells at 6, 12, 18, and 24 h; the effect peaked at 24 h for all concentrations tested (Fig. 2). SH-SY5Y cell viability was reduced to 50% at 18 h by 75 µM 6-OHDA (*P* *<* 0.001), so this concentration was chosen for subsequent experiments.

### hucMSCs contributed to 6-OHDA-stimulated SH-SY5Y cell proliferation, and Exos are involved in the process

**Fig. 3:**
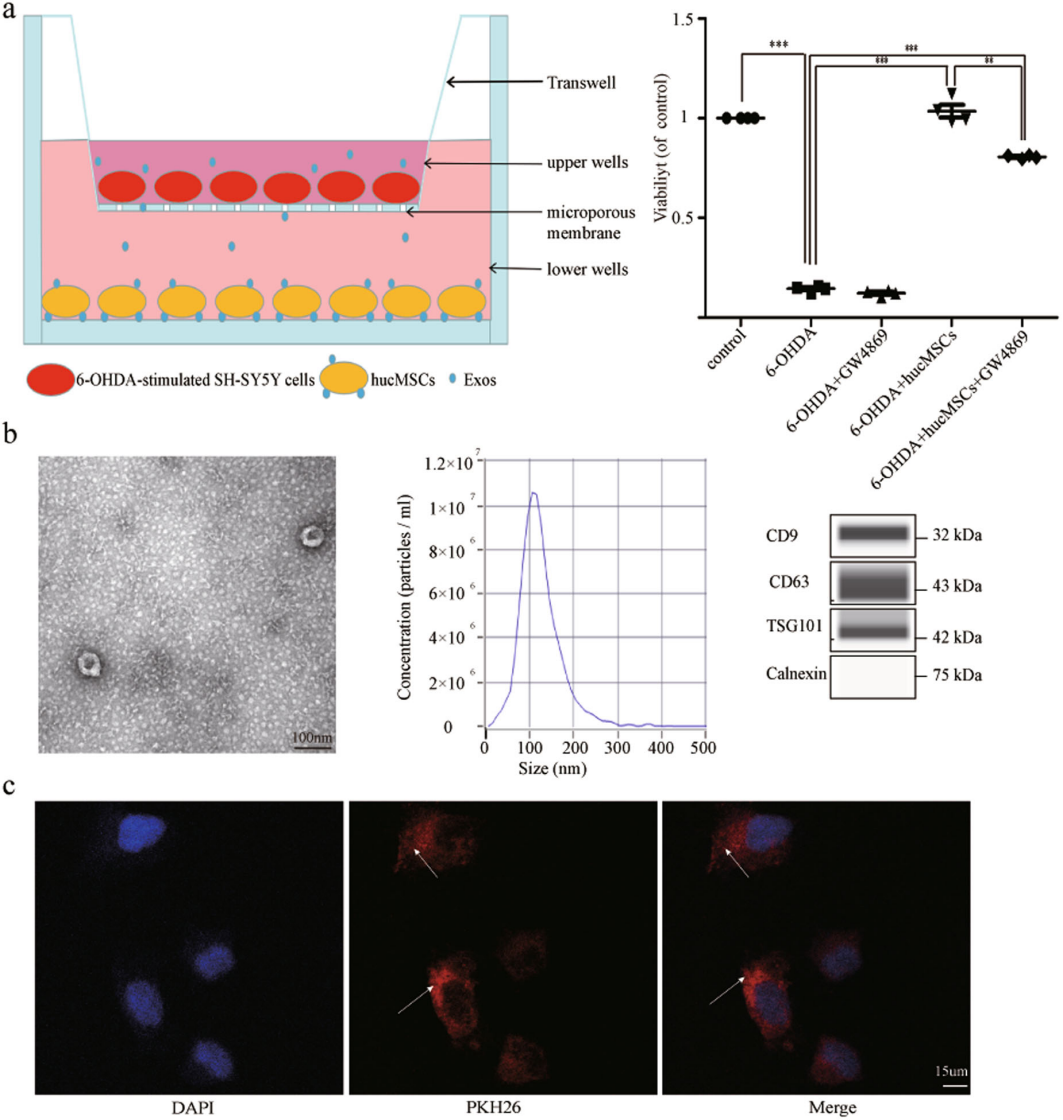
The function and typical characteristics of Exos. **a** The transwell system. hucMSCs and 6-OHDA-stimulated SH-SY5Y cells were co-cultured in a transwell system. GW4869 was used at a concentration of 10 µM to reduce the release of Exos from hucMSCs. After co-culturing for 8 h, the viability of 6-OHDA-stimulated SH-SY5Y cells was determined (*n* = 4). **b** Representative transmission electron microscopy (TEM) images of Exos. Scale bar, 100 nm. Size distributions of Exos were measured using nanoparticle tracking analysis (NTA). Western blotting analysis of Exos markers including CD9, CD63, Tsg101, and Calnexin. **c** PKH26-labeled Exos (red) and DAPI (blue). Arrows indicate Exos. All data represent mean values ± SD. *P < 0.05; **P < 0.01; ***P < 0.001.

To clarify whether Exos are involved in the process of hucMSCs therapy, we co-cultured hucMSCs with 6-OHDA-stimulated SH-SY5Y cells in a transwell system, which allows the transfer of both cytokines and Exos but prevents direct cell contact and the transfer of vesicles larger than Exos (Fig. 3a, left). GW4869, an inhibitor of Exo secretion, was used to reduce the release of Exos from hucMSCs^19^. We showed that hucMSCs activated 6-OHDA-stimulated SH-SY5Y cell proliferation and that GW4869 attenuated this effect (both *P* *<* 0.01) (Fig. 3a, right). These data suggest that hucMSCs contribute to 6-OHDA-stimulated SH-SY5Ycell proliferation and that Exos might be involved in this process.

### Exos identification and taken-up

Exos were isolated from the cultured media of hucMSCs. Fig. 3b shows a representative transmission electron microscopy (TEM) image of Exos. The diameter of Exos was found to be 30–150 nm, and the concentration and size distribution of the particles was defined by nanoparticle tracking analysis (NTA; Fig. 3b, center). Western blotting analyses indicated that Exos expressed exosomal markers such as CD9, CD63, and TSG101 protein, but did not express calnexin protein, indicating their purity (Fig. 3b, right). These results indicate that Exos were successfully purified, so these were used for subsequent studies.

To verify whether Exos could be taken-up by SH-SY5Y cells, PKH26 was used for Exos labeling, then these were co-cultured with SH-SY5Y cells. Laser scanning confocal microscopy was used to demonstrate that labeled Exos were taken-up by SH-SY5Y cells (Fig. 3c).

### Exos enhanced SH-SY5Y cell viability and reduced apoptosis

**Fig. 4:**
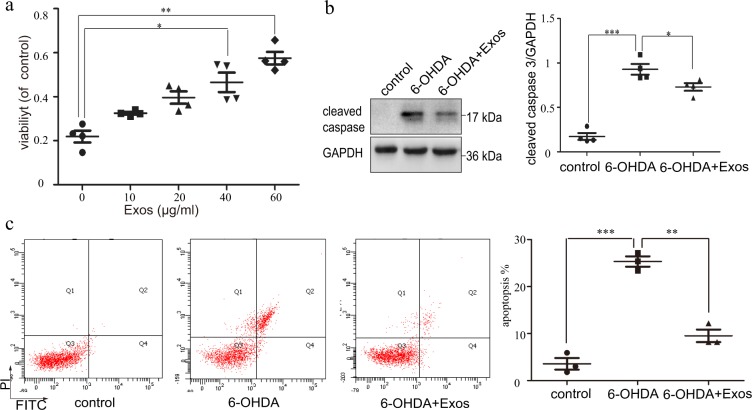
Exos enhanced SH-SY5Y cell viability and reduced SH-SY5Y cell apoptosis. **a** Cell viability was assessed by CCK-8, *n* = 4. **b** Western blotting was used to detect 6-OHDA-induced changes in apoptosis-related proteins, *n* = 4. **c** 6-OHDA-stimulated SH-SY5Y cell apoptosis was tested by FCM. All data represent mean values ± SD. *n* = 3. *P < 0.05; **P < 0.01; ***P < 0.001.

To further investigate the function of Exos, we explored their optimal intervention concentration. Cell activity increased gradually with the increase of Exos concentration and was significantly enhanced at 40 µg/ml (*P* *<* 0.05), so this was adopted for subsequent experiments (Fig. 4a). Compared with the 6-OHDA group, the expression of apoptosis-related protein (cleaved caspase-3) was significantly decreased in the 6-OHDA+Exos group (*P* *<* 0.05, Fig. 4b), as detected by western blotting. Similar results were obtained by FCM, and cell apoptosis ratio was significantly reduced in the 6-OHDA+Exos group (*P* *<* 0.01, Fig. 4c), indicating that Exos can reduce apoptosis induced by 6-OHDA.

### Exos increased the level of 6-OHDA-stimulated SH-SY5Y cell autophagy

**Fig. 5:**
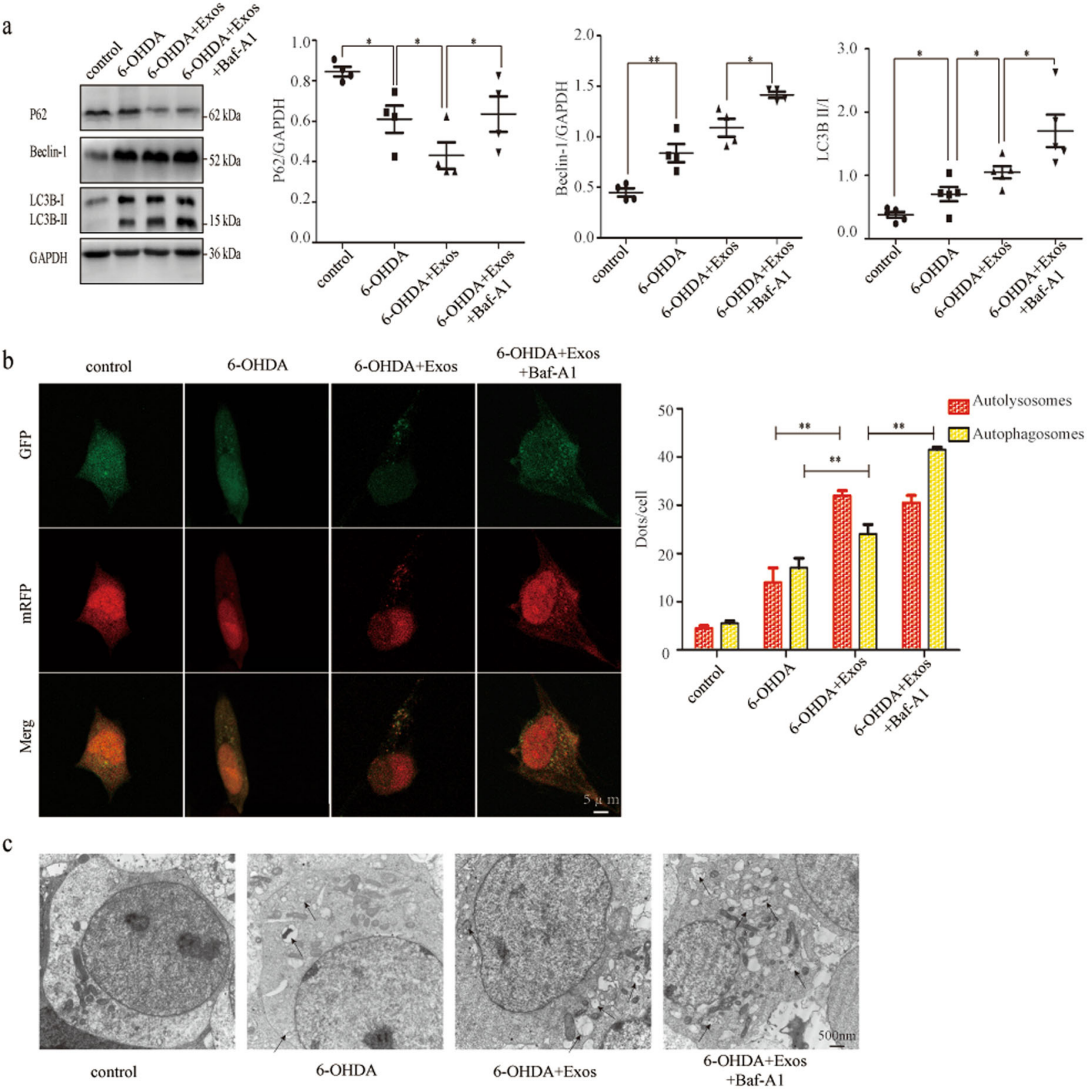
Exos increased the level of 6-OHDA-stimulated SH-SY5Y cell autophagy. **a** Western blotting was used to detect 6-OHDA-induced changes in autophagy proteins. **b** Autolysosomes and autophagosomes were detected by tandem fluorescent GFP-RFG- LC3 assay. **c** Autophagosomes were measured by TEM in four groups. All data represent mean values ± SD. *P < 0.05; **P < 0.01 (*n* = 4).

To demonstrate whether Exos induce autophagy, we detected autophagy-related proteins by western blotting. Compared with the 6-OHDA group, the expression of LC3B-II/I and Beclin-1 was significantly enhanced (both *P* *<* 0.05) and the expression of p62 was decreased in the 6-OHDA+Exos group (Fig. 5a).

To assess autophagic flux, we used the inhibitor bafilomycin A1 (Baf-A1).Compared with the 6-OHDA+Exos group, the levels of LC3B-II/I, Beclin-1, and p62 were significantly higher in the 6-OHDA+Exos+Baf-A1 group (all *P* < 0.05, Fig. 5a), suggesting that Exos activate autophagy in 6-OHDA-stimulated SH-SY5Y cells.

Additionally, we transfected adenovirus encoding the GFP-RFP-LC3 fusion gene into 6-OHDA-stimulated SH-SY5Y cells, and used laser scanning confocal microscopy to observe autophagosomes and autolysosomes. Autophagosomes were visible as yellow dots (both GFP- and RFP-positive), while autolysosomes were seen as red dots (only RFP-positive). Exos treatment significantly increased the numbers of both yellow and red dots (*P* *<* 0.01), Baf-A1 further enhanced the number of Exos-increased yellow dots (*P* *<* 0.01), whereas the number of Exos-increased red dots did not change significantly (*P* > 0.05, Fig. 5b). Autophagosomes detected by TEM were similar to those detected by double fluorescent GFP-RFP-LC3 (Fig. 5c).

Taken together, these findings provided evidence that increased autophagy induced by Exos might provide cytoprotective effects after 6-OHDA stimulation.

### Exos can cross the BBB and reach the SN, relieved the asymmetric rotation defectin a PD rat model

**Fig. 6:**
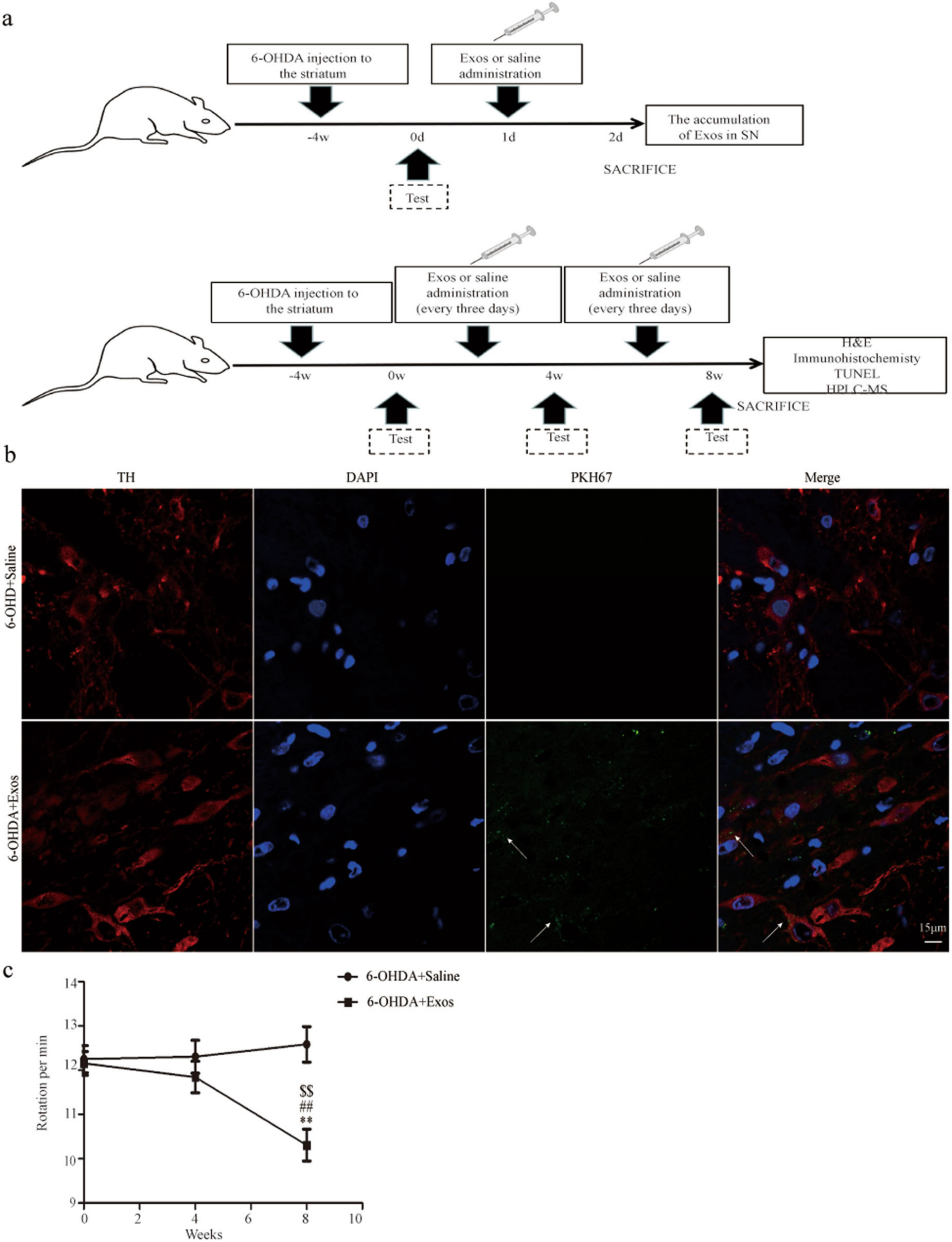
The accumulation of Exos in the SN of brain tissue in the PD rat model; Exos improved the behavioral deficits of PD model rats. **a** Experimental protocol schematic in vivo. **b** PKH67-labeled Exos were intravenously injected into rats. The co-location of Saline and Exos (green) with dopaminergic neurons in the SN of brain tissue. TH (red) indicates dopaminergic neurons. DAPI identified cell nuclei, *n* = 3. **c** APO-induced asymmetric rotation in PD model rats was relieved for 8 weeks after transplantation with Exos. APO-induced rotation in the 6-OHDA+Saline group and 6-OHDA+ Exos group. TH: Tyrosine hydroxylase. Arrows indicate Exos. All data represent mean values ± SD. **P < 0.01 vs 6-OHDA+Saline group, ^##^*P* < 0.01 vs 0 week, ^$$^*P* < 0.01 vs 4 weeks (*n* = 8).

The in vivo experimental protocol is shown in Fig. 6a. To determine whether Exos could reach the midbrain substantia nigra (SN) through the BBB, we injected saline and PKH67-labeled Exos into PD model rats. Laser scanning confocal microscopy of brain tissue showed that the green fluorescent intensity emitted by Exos was stronger and more co-localized with SN dopaminergic neurons in 6-OHDA+Exos group compared with the 6-OHDA+Saline group (Fig. 6b).

To observe the effects of Exos on a PD rat model, we treated it with saline or Exos. The apomorphine (APO)-induced asymmetric rotation was assessed at weeks 4 and 8 after the first day of transplantation. Compared with the 6-OHDA+Saline group, animals injected with Exos showed a significantly progressive reduction in contralateral rotations over time. Compared with before transplantation and 4 weeks, a significant recovery in rotation in the 6-OHDA+Exos group was observed during the 8 weeks after transplantation (*P* *<* 0.01; Fig. 6c).

These data suggest that Exos can cross the BBB and reach the SN, thereby improving the behavioral deficits of PD model rats.

### Exos significantly reduced SN dopaminergic neuron loss in a PD rat model

**Fig. 7:**
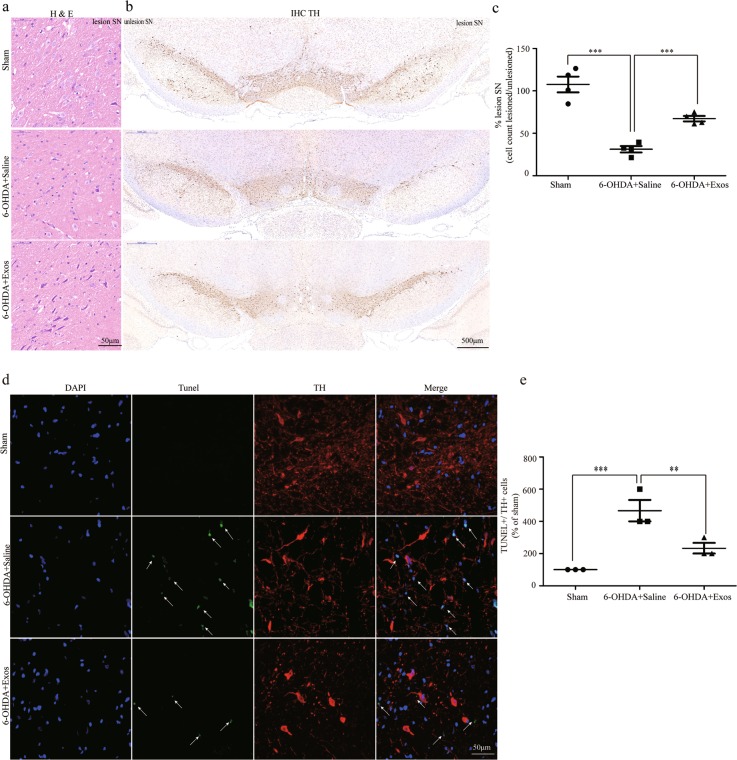
Exos significantly reduced SN dopaminergic neurons loss and apoptosis in the PD rat model. **a** Detection of tissue damage in the SN lesioned side by H&E staining. **b** SN sections from each rat were analyzed for the dopaminergic neuron counts by TH immunohistochemistry. Ventral midbrain images show the 6-OHDA lesioned side (right) compared with the unlesioned side (left). **c** Lesion severity assessed by dopaminergic neurons counts was significantly reduced in the 6-OHDA+ Exos group. **d** Representative SN section stained for the dopaminergic neuronal marker TH (red) and subjected to TUNEL assay (green) to visualize apoptosis. Nuclei of the cells are counterstained with DAPI (blue). Images were collected on the fluorescence microscopy. The arrows show significantly increased expression of TUNEL staining in the nucleus of neurons. **e** Quantitative analysis of Exos-induced protection as measured by TUNEL+/TH+ staining produced a significant neuroprotective effect. All data represent mean values ± SD. ***P < 0.001, **P < 0.01 (*n* = 4).

The examination of SN sections stained with haematoxylin and eosin (H&E) showed that pars compacta cells contained scattered large multipolar neurons with vesicular nuclei and surrounding neuropil in the sham group (Fig. 7a, top). Cells from the 6-OHDA+Saline group appeared widely distorted, angulated, and shrunken, with vacuoles in intercellular spaces. There were also different signs of degenerative and necrotic changes detected in the form of deeply eosinophilic cytoplasms, accompanied by nuclear changes in the form of pyknosis and karyolysis (Fig. 7a, middle). Treatment with Exos resulted in a significant improvement, with SN pars compacta showing multipolar neurons with nucleoli and basophilic granular cytoplasms (Fig. 7a, bottom).

SN sections from each rat were analyzed by Tyrosine hydroxylase (TH) immunohistochemistry. Ventral midbrain images showed the 6-OHDA lesioned side (right) compared with the unlesioned side (left). In the sham group, no significant difference in bilateral dopaminergic neurons was observed (Fig. 7b, top). The 6-OHDA+Saline group exhibited the greatest lesion severity (Fig. 7b, middle), while the 6-OHDA+Exos group had a significant reduction in lesion severity relative to the 6-OHDA+Saline group (Fig. 7b, bottom). Lesion severity assessed by dopaminergic neuron counting following TH staining was significantly decreased in the SN of rats given Exos but not in those receiving saline (*P* < 0.001, Fig. 7c).

These data suggest that Exos have a significant neuroprotective effect on SN dopaminergic neurons.

### Exos decreased the number of TUNEL+/TH+ cells in a PD rat model

To confirm the effect of Exos on dopaminergic neuron apoptosis, we examined SN of PD rats using TUNEL and TH double labeling. As shown in Fig. 7d, e, very few TUNEL+ cells were observed in sham group, the number of TUNEL+/TH+ cells was significantly decreased in the 6-OHDA+Exos group but not in those given saline (*P* < 0.01). Altogether, these results indicate that Exos has been shown to protect dopaminergic neuron from 6-OHDA-induced apoptosis.

### Exos upregulated the levels of dopamine and its metabolites in the striatum of PD rats

**Fig. 8:**
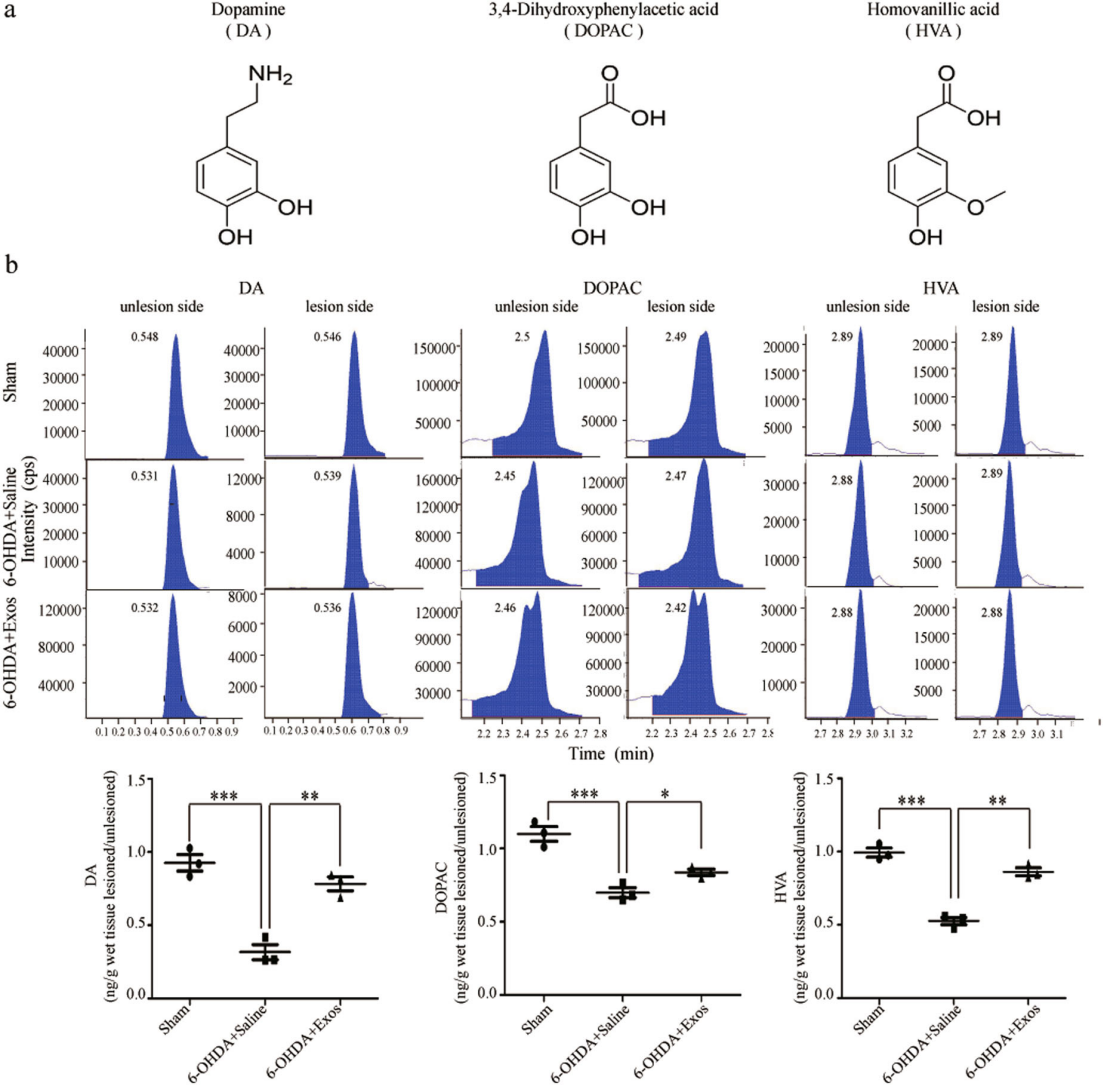
Exos upregulated the levels of DA and its metabolites in the striatum of the PD rat model. **a** Chemical structures of the analyte. **b** HPLC-MS was used to measure DA, DOPAC, and HVA levels. Exos significantly reversed 6-OHDA-downregulated DA, DOPAC, and HVA levels. All data represent mean values ± SD. *P < 0.05; **P < 0.01; ***P < 0.001 (*n* = 3).

The chemical structures of dopamine (DA) and its metabolites (Dihydroxy Phenyl Acetic Acid (DOPAC) and Homovanillic Acid (HVA)) are shown in Fig. 8a. In the present study, their levels in the striatum were measured using high-performance liquid chromatography–mass spectrometric (HPLC–MS). Compared with the sham group, the levels of DA, DOPAC, and HVA were significantly decreased in the 6-OHDA+group (all *P* *<* 0.001). After Exos administration, the levels of DA (*P* *<* 0.01), DOPAC (*P* *<* 0.05), and HVA (*P* *<* 0.01) significantly increased (Fig. 8b). These data suggest that Exos increase levels of DA and its metabolites in the striatum.

## Discussion

The findings of the present study provide new insights into the therapeutic effect of hucMSCs-derived Exos after 6-OHDA exposure in SY5Y cells and SD rats, indicating the important role of autophagy in mediating the beneficial effects of Exos on PD.

Cells typically communicate with each other through direct interaction and/or by secreting growth factors, trophic factors, and cytokines. These can act on the cell itself (autocrine signaling) or have an impact on both neighboring (paracrine signaling) and distant cells (endocrine signaling)^20^. In recent years, Exos have become recognized as potent vehicles of intercellular communication, being secreted by virtually all cell types and able to be isolated from conditioned cell media or bodily fluids^21^. While all these Exos share an evolutionary conserved set of proteins molecules including tetraspanins, Alix and Tsg101 suggesting that they share similar biological activities, they also have unique cell type-specific proteins that reflect their cellular source origin, and thus unique biological activities^22,23^. MSCs have anti-inflammatory, immune regulatory, anti-apoptosis, and microvascular regeneration effects, several studies have confirmed that MSCs have significant therapeutic effects in PD^24^. At present, the use of MSCs-derived Exos to treat PD is in the early stages. A study found that Exos secreted by stem cells derived from the dental pulp of human exfoliated deciduous teeth reduce 80% of 6-OHDA-induced dopaminergic neuron apoptosis^25^. Furthermore, Bárbara et al.^26^ have demonstrated that the reduction of dopaminergic neuronal loss observed attributes to the secreted molecules from Bone marrow MSCs (BMMSCs) rather than the transplanted BMMSCs. We chose Exos derived from hucMSCs rather than BMMSCs because hucMSCs display higher proliferation capability and faster self-renewal than BMMSCs^27^.

In the present study, we co-cultured hucMSCs with 6-OHDA-stimulated SH-SY5Y cells in a transwell system, and found that hucMSCs secretion contributed to 6-OHDA-stimulated SH-SY5Y cell proliferation. Analysis of this revealed that the therapeutic factor in the secretion is a membrane vesicle known as an Exo. We therefore hypothesized whether direct administration of Exos derived from hucMSCs may overcome the limitations and challenges associated with transplanted stem cell therapy and protect dopaminergic neurons in PD.

We speculated that Exos have effect on 6-OHDA-stimulated SH-SY5Y cell proliferation and apoptosis. In this study, Exos were obtained from the conditioned culture medium of hucMSCs. The result has shown that cell viability increased, the expression of apoptosis-related proteins and apoptotic rates were significantly decreased in group 6-OHDA+Exos. These results indicate that the Exos might provide cytoprotective effects after 6-OHDA stimulation.

Autophagy is an evolutionarily conserved and multistep lysosomal degradation process that degrades some long-lived proteins or damaged organelles in cells^28,29^. Its dysregulation may impede the clearance of abnormal proteins and damaged organelles, which is identified as part of PD pathogenesis^30,31^. Therefore, we speculate that Exos may play a role by regulating autophagy. The results indicate that the expression of autophagy-related proteins was increased in the 6-OHDA group, Exos could further increase the expression ofit. While autophagy is a dynamic and multistep process. The upregulation of autophagy-related proteins results from the activation of autophagy or the accumulation of autophagosomes caused by late autophagy inhibition. To assess autophagic flux and to distinguish autophagy induction from autophagy inhibition, we used the inhibitor bafilomycin A1 (Baf-A1) which prevents autophagosome degradation by inhibiting the vacuolar type H+-ATPase^32^. We found that the autophagy-related proteins were significantly higher in the 6-OHDA+Exos+Baf-A1 group, suggesting that Exos do act by inducing autophagy. In order to observe the number of autophagosomes and autolysosomes more intuitively, the laser scanning confocal microscopy and the TEM were used. The findings were similar to western blotting. Taken together, these findings provided evidence that increased autophagy induced by Exos might provide cytoprotective effects after 6-OHDA stimulation. However, the concept of selective autophagy has now been extended with the description of precision autophagy, so the point of intervention in the autophagy pathway might be an important consideration. Despite great advances in our understanding of how Exos could repair the PD model injury by inducing autophagy, specific knowledge of the events taking place remains limited. We will study this further in future experiments.

Because Exos are widely found in humans, they are thought to have low immunogenicity and a long circulating half-life^33,34^. Furthermore, some peptides transported by Exos specifically combine with receptors in targeted cells, accelerating the accumulation of Exos in targeted tissues^35^. Therefore, Exos show a targeted ability when grafted through the ligand receptor-mediated active targeting process. The existence of the BBB makes it difficult for drugs to reach lesion sites, thus affecting therapeutic effects in central nervous system diseases. The previous research has shown that Exos can cross the BBB^36^, the present study also have come to the same conclusion that Exos can reach the SN. This greatly overcomes the limitation of low BBB permeability of stem cells.

PD rats induced by 6-OHDA can produce pathological and biochemical manifestations similar to human PD, including progressive degeneration and death of dopaminergic neurons in SN pars compacta, glial cell proliferation and decrease of DA level in the striatum, and then there will be the characteristic motor dysfunction, including resting tremor, bradykinesia, rigidity, and postural instability^37^. Therefore, we evaluated the therapeutic effect of Exos on PD rats based on the above mechanisms and manifestations.

In many studies the two rotation-inducing agents D-amphetamine and APO have been used to behaviorally assay the extent of neuronal loss following lesions of the substantia nigra pars compacta. However, D-amphetamine is able to induce rotation in normal rats due to a natural imbalance in dopamine between bilateral caudate–putamen complexes (CPu)^38^. Furthermore, extensive damage of dopaminergic fibers and neurons are required to generate rotations demonstrable with a low dose of APO but not with amphetamine^39^. Therefore, we used APO to induce rotation in our study. The results show that Exos relieved the asymmetric rotation defectin a PD rat model.

TH is the rate-limiting enzyme for dopamine synthesis in neurons, and the expression of TH in cells is the gold standard for dopaminergic neuron identification. SN sections from each rat were therefore analyzed by TH immunohistochemistry. The result has shown that the number of dopaminergic neurons increased significantly after Exos treatment; moreover, the number of dopaminergic neurons apoptosis decreased significantly suggest that the Exos have a neuroprotective effect on SN dopaminergic neurons. The determination of DA and its metabolites can be achieved by a variety of analytical methods including spectrophotometry^40^, spectrofluorimetry^41^, chromatographic methods^42,43^, mass spectroscopy^44^, chemiluminesence^45^, and electrophoresis^46^. However, HPLC–MS methods have attracted a great deal of interest because of their simple pre-treatments, high selectivity and sensitivity. In the present study, the levels of DA and its metabolitesin the striatum were measured using HPLC–MS. The results showed that the level of dopamine in striatum of rats treated with Exos was significantly higher than that of saline group, indicating that Exos (but not APO) increased the levels of DA and its metabolites in the striatum.

Previous studies have demonstrated that MSC-derived Exos impact the activity of targeted cells through transfering proteins, mRNAs and miRNAs. In this regard, Iglesias et al.^47^ have shown that coculture of fibroblasts and proximal tubular cells from cystinosis patients with BMMSC-derived Exos resulted in a dose-dependent decrease in cellular cystine levels because BMMSC-derived Exos contain cystinosin, a cystine efflux channel in the lysosomal membrane. Tomasoni et al.^48^ have reported that the Exos derived by BMMSC contains insulin-like growth factor-1(IGF-1) receptor mRNA, which can ameliorating renal dysfunction and repair tubular damage of acute kidney injury (AKI). In addition, Exos contain miRNAs which is able to inhibit tumor growth^49,50^, reduce cardiac fibrosis following myocardial infarction^51^, stimulate axonal growth from cortical neurons^52^, promote neurite remodeling and functional recovery after stroke^53^, and stimulate endothelial cell angiogenesis^54^. For example, Yang and colleagues^55^ have proposed that BMMSC-derived Exos can prevent ovarian follicular atresia in cyclophosphamide (CTX)-treated rats via delivering miR-144-5p into ovarian cells, an effect is reversed by inhibition of miR-144-5p. Furthermore, systemic injection of miR-133b containing Exos preserves neurons, promotes axon regeneration, and improves the recovery of hindlimb locomotor function following spinal cord injury^56^. Actually, miR-133b regulates the transcriptional activator, Pitx3, an important factor in dopaminergic neurons development^57^ and is downregulated in PD patients. However, it is not clear whether the impact of MSC-derived Exos in the present study is due to miR-133b. Therefore, future studies are warranted to determine the mechanisms underlying hucMSC-derived Exos-induced neuroprotective effect.

## Materials and methods

### Cell culture and identification

Fresh umbilical cords were obtained from informed, consenting mothers and processed within 4–6 h. The cords were washed twice to remove remaining blood in warmed phosphate-buffered saline (PBS) supplemented with antibiotics and antimycotics. The washed cords were cut longitudinally with a sterile scalpel to expose Wharton’s jelly and then sliced into 0.3–0.5 cm^3^ pieces. To facilitate the attachment of the pieces to the plastic surface, they were left exposed to the air for 5 min before adding 15 ml of cell culture medium. This consisted of low-glucose Dulbecco’s modified Eagle medium (DMEM; Gibco, CA, USA) supplemented with 10% fetal bovine serum (FBS; Gibco).The pieces of cord were then cultured at 37 °C in humid air with 5% CO_2_. The medium was replaced every 3 days after the initial plating. When confluency had reached 80%, the cells were passaged into new flasks for further expansion.

The proliferation of cells was measured using a HoloMonitor® time-lapse cytometer (BIO-SUN SCI&TECH, Beijing, China). Cells at passage 3 were seeded into 6-well plates at 1.3 × 10^5^ cells per well. They were observed for 6 days, and the medium was replaced after 3 days. FCM was performed to detect marker expression using the following fluorescein isothiocyanate (FITC)-conjugated or phycoerythrin-conjugated antibodies: CD73, CD45, CD34, CD90, CD105, and HLA-DR (BD Biosciences®, Sparks, MD, USA).

We also evaluated the multidirectional differentiation potential of cells. Cells at passage 3 were seeded into 24-well plates at 6 × 10^4^ cells per well. The next day, it was confirmed that the degree of cell fusion had reached 80–90%, after which the medium was changed to osteogenic differentiation medium, adipogenic differentiation medium, or chondrogenic differentiation medium (Biological Industries, Beit-Haemek, Israel). The medium was replaced every 2–3 days. After 3 weeks, the osteogenic differentiation of the cells was examined by Alizarin Red S staining, adipocytes were stained with Oil Red O, and chondrocytes were stained with Alcian Blue 8GX (Biological Industries).

SH-SY5Y cell lines were kindly provided and authenticated by Stem Cell Bank, Chinese Academy of Sciences. cultured in DMEM: Nutrient Mixture F-12 (DMEM/F-12) containing 10% FBS at 37 °C in a humid environment containing 5% CO_2_.

### Coculture of 6-OHDA-stimulated SH-SY5Y cells and hucMSCs

To clarify whether Exos are involved in neuroprotection provided by hucMSCs, the following groups were established: the control group, the 6-OHDA group, the 6-OHDA+GW4869 group, the 6-OHDA+ hucMSCs group, and the 6-OHDA+ hucMSCs+ GW4869 group. SH-SY5Y cells were co-cultured with hucMSCs in a Transwell-24 system with a 0.4 µm porous membrane (Corning®, Tewksbury, MA, USA) to prevent both the transfer of vesicles larger than Exos and direct cell contact for 12 h. They were then incubated with 75 μΜ 6-OHDA for 18 h, after which cell viability was detected. hucMSCs were seeded in the lower wells and SH-SY5Y cells were planted in the upper wells. GW4869 (Sigma-Aldrich,St. Louis, MO, USA) was used at a concentration of 10 µM to reduce the release of Exos from hucMSCs^19^. Before hucMSCs were co-cultured with SH-SY5Y cells, they were stimulated with GW4869 for 8 h.

### Exos purification, identification, and tracking

hucMSCs were cultured for 48h in serum-free medium, then the culture medium was collected in a centrifuge tube. The choice of this time is mainly depending on the speed of division of cells, which varies greatly with the cell type^58^. We found in our pilot study that the cell density were profoundly increased after 48h culture, which may affect the secretion of Exos. Moreover, the best storage of Exos is at 4 °C or −80 °C, Exos secreted in the previous 48 h can also be damaged if left in cell culture for too long, so we collected the supernatant of cells in 48 h. This was centrifuged for 20 min at 2000 × *g* at 4 °C to eliminate debris. The supernatant was transferred to a polycarbonate tube suitable for use in ultracentrifuge rotors, which was marked at the bottom towards the outside of the rotor to help locate the pellet. Tubes were centrifuged for 30 min at 10,000 × *g* at 4 °C, then the supernatant was collected without contaminating it with the pellet. The supernatant was centrifuged again for 70 min at 100,000 × *g* at 4 °C, the new supernatant was removed, and the pellet was resuspended in PBS. This was again centrifuged for 70 min at 100,000 × *g* at 4 °C, and the pellet was resuspended in PBS and stored at –80 °C. The Exos concentration was analyzed by the BCA method (Solarbio, Beijing, China).

The ultrastructure and size distribution of Exos were analyzed by TEM (H-7500, Hitachi, Tokyo, Japan) and Nanosight (Malvern, Malvern, UK) respectively. The expression of protein markers was analyzed by western blotting using antibodies against CD9 (dilution 1:500, ab2215, Abcam, Cambridge, UK), CD63 (dilution 1:1000, ab59479, Abcam), TSG101 (dilution 1:500, ab83, Abcam), and calnexin (dilution 1:1000, 2679T, Cell Signaling Technology, MA, USA). The above identification meet the minimum identification requirements for the study of vesicles issued by the International society of extracellular vesicles^59^.

For up-take studies, purified Exos were labeled with a PKH26 kit (Sigma-Aldrich) according to the manufacturer’s protocol. Briefly, the Exos pellet was resuspended in 1 ml Diluent C, while in parallel 4 µl PKH26 dye was added to 1 ml Diluent C and incubated with the Exos solution for 4 min at room temperature. Then 2 ml FBS was added to bind excess dye. Labeled Exos were collected by centrifuging at 100,000 × *g* for 1 h, then the Exos pellet was resuspended in serum-free medium and co-cultured with SH-SY5Y cells for 12 h, fixed, DAPI staining and visualized with laser scanning confocal microscopy (Olympus®, Tokyo, Japan).

### Cell viability assay

The CCK-8 kit (Solarbio) was used to measure SH-SY5Y cell viability. Cells (3 × 10^4^/well) were seeded in 96-well plates overnight. To detect the negative effects of 6-OHDA (Sigma-Aldrich) on SH-SY5Y cell viability, cells were incubated with different concentrations (50, 75, 100, 125, and 150 µM) of 6-OHDA for 6, 12, 18, and 24 h. Normal culture media were used for the control group.

To detect the beneficial effects of Exos on SH-SY5Y cell viability, SH-SY5Y cells were first co-cultured with different concentrations (0, 10, 20, 40, and 80 µg/ml) of Exos for 12 h and then exposed to 6-OHDA (75 µM) for 18 h. Another group was only co-cultured with 6-OHDA (75 µM) for 18 h. Normal culture media were used for the control group.

At prespecified time points, 10 µL of CCK-8 was added to the cells and incubated for 2.5 h. Optical density values were determined at 450 nm using a microplate reader (Thermo Fisher Scientific, MA, USA). Each group was tested in quadruplicate in three replicate wells. The cell viability of experimental groups was calculated relative to that of the control group.

### Annexin V- FITC/propidium iodide (PI) apoptosis assay

To evaluate the effect of Exos on 6-OHDA-stimulated SH-SY5Y cell apoptosis, the Annexin V-FITC/PI apoptosis detection kit (BD Biosciences®, Sparks, MD, USA) was used according to the manufacturer’s protocol. A total of 1 × 10^6^ SH-SY5Y cells were seeded in 6-well plates overnight, cells in 6-OHDA+Exos group were co-cultured with Exos (40 µg/ml) for 12 h and then exposed to 6-OHDA (75 µM) for 18 h, cells in the 6-OHDA group were only co-cultured with 6-OHDA (75 µM) for 18 h. Normal culture media were used for the control group.

At prespecified time points, cells were collected, washed twice with cold PBS, and then resuspended in 1 × Binding Buffer at a concentration of 1 × 10^6^ cells/ml. A total of 100 µL of the solution (1 × 10^5^ cells) was transferred to a 5 ml culture tube, and 5 µL of FITC Annexin V and 5 µL PI were added. Cells were gently vortexed and incubated for 15 min at 25 °C in the dark. Then, 400 µL of 1 × Binding Buffer was added to each tube and apoptosis was detected by FCM (FC500; Beckman Coulter, Miami, FL, USA) and analyzed by CytoDiff CXP 2.0 software (Beckman Coulter). Experiments were carried out in quadruplicate.

### Western blot analysis

Exos related proteins were detected by simple western, which is a gel-free, blot-free, capillary-based, automated protein immunodetection system that automates all the steps following sample preparation including sample loading, size-based protein separation, immunoprobing, washing, detection, and data analysis^60^. All procedures were performed with manufacturer’s reagents according to the user manual. Briefly, master mix and protein sample 1:4 ratio fully mixed, vortexed, and heated at 95 °C for 5 min The samples, blocking reagent, wash buffer, primary antibodies, secondary antibodies, and chemiluminescent substrate were dispensed into designated wells in the manufacturer-provided microplate. Following plate loading, separation and immunodetection were performed automatically using default settings. Data were analyzed with Compass software (ProteinSimple).

To detect the expression levels of cleaved caspase 3, an apoptosis-related protein, western blotting was performed using an antibody against cleaved caspase 3 (dilution 1:800, 19677-1-AP, Proteintech, Wuhan, China). To detect the expression of autophagy-related proteins, the following antibodies were used: Beclin-1 (dilution 1:1000, 11306-1-AP, Proteintech), LC3B (dilution 1:1000, 14600-1-AP, Proteintech), and p62 (dilution 1:2000, 18420-1-AP, Proteintech). To detect changes in the autophagic flux, SH-SY5Y cells were co-cultured with Exos for 12 h, then 100 nM Baf-A1 (Med Chem Express) was added to the cells 1;h before exposure to 6-OHDA (75 µM) for 18 h; the three other groups were treated as annexin V- FITC/PI apoptosis assay described.

At prespecified time points, cells were collected and protein concentrations were determined with a BCA kit (Solarbio). An equal quantity of protein (20–30 µg) from each sample was subjected to electrophoresis and transferred to a polyvinylidene difluoride membrane (Millipore). The membrane was blocked with 5% milk in Tris-buffered saline with Tween 20 for 2 h and then incubated overnight with primary antibodies. Then, it was incubated with secondary antibodies for 1 h, and stained with enhanced chemiluminescence (ECL kit, Millipore). The relative levels of immunoreactivity were analyzed by densitometry with Image J Software (NIH) and were normalized to a loading control (GAPDH; dilution 1:20000, 10494-1-AP, Proteintech).

### Autophagic flux measurements

To detect autophagic flux, the mRFP-GFP-LC3 reporter plasmid (Hanbio, Shanghai, China) was transfected into SH-SY5Y cells according to the manufacturer’s instructions. Transfected cells were processed and grouped as described above. Cell images were obtained using a laser scanning confocal microscopy, and autophagosome and autolysosome dots were quantified manually in at least four different SH-SY5Y cells per group.

### Transmission electron microscopy

To evaluate the characteristics of the Exos, they were purified as described above. Undiluted Exos were exposed to 3% formaldehyde and 0.1% glutaraldehyde for 10 min at 37 °C. Then, Exos were loaded onto form var/carbon-coated grids, contrasted with 2% uranyl acetate and examined by TEM (HITACHI H-7650).

To measure autophagosomes, SH-SY5Y cells were seeded and grouped as described above. Cells were harvested by centrifugation at 300 × *g*, then sectioned and fixed in 4% glutaraldehyde containing 2% paraformaldehyde in 30 mM phosphate buffer, pH 7.4. After dehydration with alcohol, the slides were placed in embedding molds saturated in propylene oxide and incubated at 60 °C for 48 h. The 70-nm-thin sections were prepared and detected by TEM. A total of four images of each sample were examined in three independent experiments.

### Animals

Adult male SD rats (200–220 g) were purchased from Beijing Vital River Laboratory Animal Technology Co., Ltd. (Beijing, China; License No. SCXK (Jing) 2016-0006) and kept under specific pathogen-free conditions. A controlled environment of 20 ± 2 °C, 45–50% humidity, and a 12-h light/dark cycle was maintained throughout the study. All animal protocols were approved and conducted according to recommendations from the Research Subcommittee of Hebei Medical University on Animal Care and Use and the Chinese Council on Animal Care.

### The 6-OHDA injury model and animal grouping

Stereotaxic injection with 6-OHDAon the right striatum was applied to SD rats to establish an animal model of PD. Rats were anaesthetized with 2% isoflurane and placed in the stereotaxic apparatus (Neurostar, Tubingen, Germany). For 6-OHDA intoxication, rats were stereotaxically injected with 6-OHDA (15 µg in 3 μl of saline with 0.2% ascorbic acid) at the same insertion point and different depths in the right striatum with the following coordinates: AP –0.2 mm, ML –3 mm, DV –4.5 mm; AP –0.2 mm, ML –3 mm, DV –5.5 mm (coordinates were based on the stereotaxic map of the brain 1993^61^). The sham group was injected with the same dose of saline with 0.2% ascorbic acid (*n* = 12). After each injection, the needle was left in place for 10 min before it was withdrawn slowly to facilitate toxin infusion and to prevent reflux. After surgery, rats were kept in individual cages.

APO-induced rotation is as an ideal predictor of maximal unilateral striatum dopamine depletions^61^. Before and 4 weeks after surgery, rats received i.p. injection of 0.5 mg/kg APO. After they were acclimated for 5 min, rotational data were continuously recorded for 30 min. Rats with >7 turns/min were considered to be successful models^62–68^.

Rats successfully modeled were randomly assigned in equal numbers to two groups (*n* = 12 per group) as follows: (1) the 6-OHDA+Saline group, in which 0.5 ml saline per rat was administered over 5 min via the tail vein; and (2) the 6-OHDA+Exos group, in which 200 µg total protein of Exos precipitated in an equal volume of saline (0.5 ml) was administered over 5 min via the tail vein. Rats in both groups were administered every 3 days for 8 weeks. This protocol was chosen based on our pretest results.

### Co-location of Exos with dopaminergic neurons in the SN

To verify whether Exos can reach the SN through the BBB, PD rats were randomly divided into two groups (*n* = 3 per group): 6-OHDA+saline and 6-OHDA+Exos groups. The procedure for labeling Exos with PKH67 is described above. After 24 h, animals were anaesthetized and their brains were harvested and post-fixed for 1 day with 4% paraformaldehyde. They were then transferred to 10%, 20%, and 30% sucrose solutions at 4 °C, then embedded in optimal cutting temperature (OCT) compound. Brains were sectioned at 6 μm thickness using a microtome (CM1950; Leica, Heerbrugg, Switzerland). After washing with PBS, sections were incubated with 1% bovine serum albumin containing 0.3% Triton X-100 in PBS. Sections were then incubated with rabbit TH (dilution 1:500, GB11181, Servicebio, Wuhan, China) at 4 °C overnight. The next day, sections were incubated with Cy3-conjugated donkey anti–rabbit antibody (dilution 1:250, GB21403, Servicebio) at room temperature for 1 h. Each of the steps was followed by three 5-min rinses in PBS. Immunoreactive cells were visualized using the confocal laser microscope (Olympus).

### Haematoxylin and eosin (H&E) and immunohistochemistry

Brain tissue was obtained after behavioral tests for H&E staining and immunohistochemistry. Brains were dehydrated in an ascending ethanol series, embedded in paraffin and sectioned into 3 µm sections using a microtome (Leica). Sections were dewaxed by xylene (I, II) for 10 min each, rehydrated in alcohol (100%, 95%, 80%, and 70%) for 5 min each, washed with running tap water and stained in Mayer’s haematoxylin for 4 min Sections were washed and “blued” in running tap water. In the case of high background, sections were briefly differentiated in acid alcohol four times then washed in tap water, re-blued, and assessed under the microscope. Sections were then dehydrated in ethanol (85%, 95%) for 5 min each, stained with eosin solution for 5 min, and rinsed in tap water to remove excess stain. Finally they were rapidly dehydrated through graded ethanol in xylene and sealed using neutral gum.

Slice dewaxing and rehydration was performed as described above, then endogenous peroxidase was sealed with 3% hydrogen peroxide and repaired by antigen. Then, a primary antibody against TH (dilution 1:1000, GB11181, Servicebio) was added and incubated for 1 h at 4 °C overnight. The secondary antibody labeled with horseradish peroxidase (dilution 1:200, GB23303, Servicebio) was added and incubated for 30 min at 37 °C, then fresh 3, 3′-diaminobenzidine was added for colouration for 1–2 min. Slices were then washed three times with PBS for 5 min, counterstained with haematoxylin for 1 min, dehydrated, and sealed using neutral gum. Slices were observed with an optical microscope, and images were taken for analysis.

### TUNEL assay

For quantification of apoptotic in the SN, the In Situ Cell Death Detection Kit (Roche, Mannheim, Germany) was used according to the user’s manual. Brielfly, slice dewaxing step is the same as above, then use the membrane breaking working liquid to break the membrane. Subsequently, the percentage of apoptotic dopaminergic neurons was determined by double immunofluorescence staining for TUNEL and TH (1:500, Servicebio) overnight at 4 °C. Then, the slides were washed three times with PBS and incubated with the appropriate secondary antibody for 50 min at room temperature. After incubation with the DAPI, the green fluorescein-labeled DNA and red fluorescein-labeled dopaminergic neurons were visualized by fluorescence microscopy (Leica DM4000, Berlin, Germany).

### HPLC-MS analysis

Stock solution was dissolved in methanol to a concentration of 1 mg/ml. The stock solution was serially diluted to obtain final concentrations of 5, 10, 50, 100, 200, and 500 ng/ml with methanol. Using linear regression constructed a calibration curve.

Chromatographic separation was performed on an LC-30AD System (Shimadzu, Kyoto, Japan) connected to a CTC Sample Manager (Waters Corporation, Milford, MA, USA) operated at 5 °C. The mobile phase consisted of a gradient of water (A) and acetonitrile (B), both containing 0.1% formic acid. The flow rate was maintained at 300 μl/min, and the gradient profile is shown in Table 1. Separation of the analytes was performed on an XB-C18 column (Welch, 1.8 μm particle size, 50 × 2.1 mm, Welch, US) maintained at 40 °C. The injection volume was 5 μl. MS/MS detection was carried out on a Mass Spectrometer QTRAP 4500 (ABSciex, Canada) equipped with an ESI source operated in the positive ion mode. MS analysis was performed in multiple reaction monitoring mode. The dwell time was set to 300 ms. The optimized ion source parameters were curtain gas: 10 psi; collision gas: 8 psi; capillary voltage: 5500 V; nebulizer gas: 40 psi; turbo gas: 40 psi; and ion source temperature: 500 °C. Data acquisition and processing were achieved using Analyst (ABSciex, Canada).

**Table. 1:** Mobile phase gradient elution program.

Time (min)	A%	B%
0–1.0	95	5
4.0	10	90
5.2	0	100
5.2–7.0	95	5

### Statistical analysis

One-way analysis of variance was used to determine the level of significance with SPSS software, version 20.0. *P* *<* 0.05 was considered statistically significant. Samples were randomly located into different groups by random number method. The investigators were blinder to group allocation during data collection and analysis. Sample size was calculated by the formula: *n* = 2 [(*U*_α_ + *U*_β_) Sδ^−1^]^2^. *S* in the formula referred to the standard deviation (s.d.). δin the formula represented the inter-group difference of the mean values. There was a difference above 0.2 in DA(ng/g wet tissue lesioned/unlesioned) between treatment group and control group, i.e., δ = 0.2. s.d. of DA (ng/g wet tissue lesioned/unlesioned) from previous studies was 0.1, i.e., *S* = 0.1. We selected the significant level at 5% (α = 0.05) in a two-tailed test and power of the study at 90% (1 − β = 0.9). According to the formula: *n* = 2 [(*U*_α_ + *U*_β_) *S*δ^−1^]^2^, *U*_0.05_ = 1.960, β = 0.10, *U*_0.1_ = 1.282, we chose *n* = 12 for each group, which was enough to detect the real difference among groups. Animals in poor body condition or other sick conditions were excluded. The exclusion was made before random group assignments, experimental intervention and data analysis.
